# Automatic segmentation model and machine learning model grounded in ultrasound radiomics for distinguishing between low malignant risk and intermediate-high malignant risk of adnexal masses

**DOI:** 10.1186/s13244-024-01874-7

**Published:** 2025-01-13

**Authors:** Lu Liu, Wenjun Cai, Feibo Zheng, Hongyan Tian, Yanping Li, Ting Wang, Xiaonan Chen, Wenjing Zhu

**Affiliations:** 1 Department of Ultrasound Medicine, South China Hospital, Medical School, Shenzhen University, Shenzhen, P. R. China; 2 Department of Ultrasound, Shenzhen University General Hospital, Medical School, Shenzhen University, Shenzhen, P. R. China; 3 Department of Nuclear Medicine, Qingdao Hospital, University of Health and Rehabilitation Sciences (Qingdao Municipal Hospital), Qingdao, P. R. China; 4 Department of Urology, Shengjing Hospital of China Medical University, Shenyang, P. R. China; 5 Medical Research Department, Qingdao Hospital, University of Health and Rehabilitation Sciences (Qingdao Municipal Hospital), Qingdao, P. R. China

**Keywords:** Ultrasound, Segmentation, Machine learning, Deep learning, Adnexal mass

## Abstract

**Objective:**

To develop an automatic segmentation model to delineate the adnexal masses and construct a machine learning model to differentiate between low malignant risk and intermediate-high malignant risk of adnexal masses based on ovarian-adnexal reporting and data system (O-RADS).

**Methods:**

A total of 663 ultrasound images of adnexal mass were collected and divided into two sets according to experienced radiologists: a low malignant risk set (*n* = 446) and an intermediate-high malignant risk set (*n* = 217). Deep learning segmentation models were trained and selected to automatically segment adnexal masses. Radiomics features were extracted utilizing a feature analysis system in Pyradiomics. Feature selection was conducted using the Spearman correlation analysis, Mann–Whitney *U*-test, and least absolute shrinkage and selection operator (LASSO) regression. A nomogram integrating radiomic and clinical features using a machine learning model was established and evaluated. The SHapley Additive exPlanations were used for model interpretability and visualization.

**Results:**

The FCN ResNet101 demonstrated the highest segmentation performance for adnexal masses (Dice similarity coefficient: 89.1%). Support vector machine achieved the best AUC (0.961, 95% CI: 0.925–0.996). The nomogram using the LightGBM algorithm reached the best AUC (0.966, 95% CI: 0.927–1.000). The diagnostic performance of the nomogram was comparable to that of experienced radiologists (*p* > 0.05) and outperformed that of less-experienced radiologists (*p* < 0.05). The model significantly improved the diagnostic accuracy of less-experienced radiologists.

**Conclusions:**

The segmentation model serves as a valuable tool for the automated delineation of adnexal lesions. The machine learning model exhibited commendable classification capability and outperformed the diagnostic performance of less-experienced radiologists.

**Critical relevance statement:**

The ultrasound radiomics-based machine learning model holds the potential to elevate the professional ability of less-experienced radiologists and can be used to assist in the clinical screening of ovarian cancer.

**Key Points:**

- We developed an image segmentation model to automatically delineate adnexal masses.
- We developed a model to classify adnexal masses based on O-RADS.
- The machine learning model has achieved commendable classification performance.
- The machine learning model possesses the capability to enhance the proficiency of less-experienced radiologists.
- We used SHapley Additive exPlanations to interpret and visualize the model.

**Graphical Abstract:**

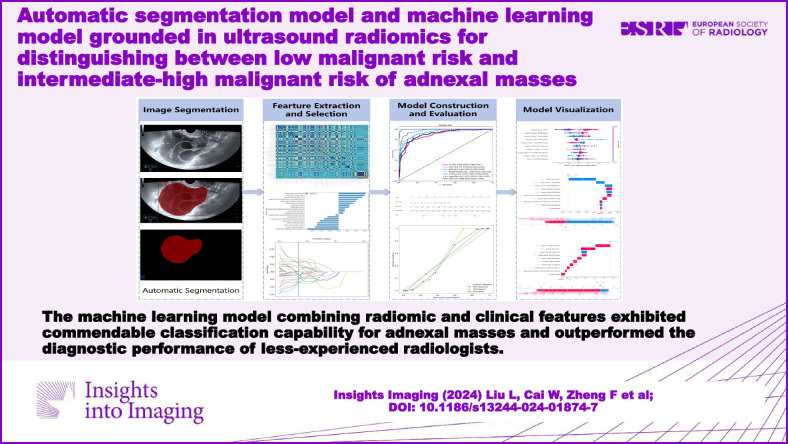

## Introduction

Adnexal masses encompass ovarian, fallopian tube, and para-ovarian masses, representing a varied spectrum of benign, malignant, and borderline conditions [1]. The prognosis markedly differs according to different histopathological types [2]. Notably, ovarian cancer represents the deadliest gynecological tumor [3], with a five-year relative survival rate of less than 50% [4].

The therapeutic approach for malignant and benign adnexal masses is fundamentally distinct [5, 6]. Consequently, precise differentiation between malignant and benign lesions is critical for optimizing patient care. Ultrasound currently stands as the primary imaging modality for adnexal lesions. Considering the diverse morphological features presented by these masses, interpreting ultrasound images can be intricate, with diagnostic accuracy heavily dependent on the radiologist’s experience and subjective assessment.

To facilitate the evaluation process for adnexal lesions, several evidence-based risk stratification systems have been developed [7–13]. The ovarian-adnexal reporting and data system (O-RADS) risk stratification and management system categorizes adnexal masses into 6 types, ranging from normal to high risk of malignancy [14, 15]. The adoption of O-RADS in clinical practice is increasingly widespread, and its diagnostic efficacy in differentiating between malignant and benign masses has been validated [16–21]. However, the complexity of the system and the varied presentation of adnexal masses pose a challenge for radiologists, particularly those with less experience.

Recently, artificial intelligence (AI)—aided image analysis has facilitated more precise and consistent evaluations of adnexal lesions [22]. To enhance ultrasound screening for ovarian cancer and assist less-experienced radiologists in improving their professional skills, we established a segmentation model to delineate the adnexal masses. Additionally, we constructed an ultrasound radiomics-based machine learning model integrating radiomic and clinical features to differentiate between low malignant risk and intermediate-high malignant risk adnexal masses based on O-RADS. The SHapley Additive exPlanations (SHAP) method was used for model interpretability and visualization.

## Materials and methods

### Ethical approval

**Fig. 1:**
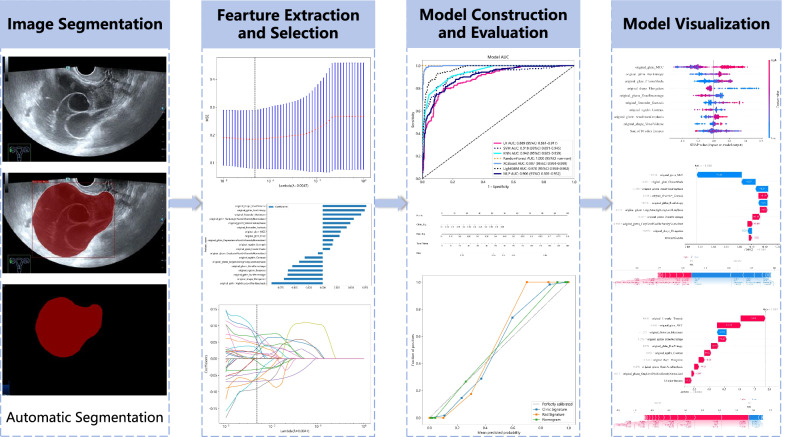
Workflow of ultrasound-based radiomics analysis

The research was authorized by the ethics committees of South China Hospital of Shenzhen University (no.: HNLS20230112101-A). Considering the retrospective nature of the research, a waiver for patient informed consent was provided. The workflow for model construction and visualization is illustrated in Fig. 1.

### Participants and data collection

From June 2021 to January 2024, we conducted a retrospective collection of ultrasound images (through the vagina or rectal) from participants diagnosed with adnexal masses at the South China Hospital of Shenzhen University (training set) and the Qingdao Municipal Hospital (testing set). In adherence to O-RADS, two experienced radiologists (H.T. and W.C.) with two decades of gynecological ultrasound experience categorized these images into five types (O-RADS 1–5), and their consistent classification served as the diagnostic criterion. Given that O-RADS 4 has been established as the optimal threshold for malignancy [16, 18], the masses were divided into two subsets: O-RADS 1–3 lesions were considered a low malignant risk set, whereas O-RADS 4–5 lesions were considered an intermediate-high malignant risk set. In cases of disagreement, a gynecological ultrasound specialist would be consulted to reach a consensus.

The participants’ clinical features included age, the mass’ maximum diameter, symptoms, menopausal status, and whether ascites were present or absent. The symptoms encompassed dysmenorrhea, chronic pelvic pain, abdominal discomfort, dyspareunia, and a sensation of abdominal distension.

The eligibility criteria included: (1) participants with adnexal lesions had undergone ultrasound examination at the two designated hospitals mentioned above; (2) participants over the age of 18; and (3) participants with multiple adnexal masses, only the mass exhibiting the most intricate morphology were included. The exclusion criteria included: (1) pelvic masses that could not be confirmed as originating from the adnexa; (2) low-quality images; and (3) incomplete clinical data.

### Image acquisition

The ultrasonic examination was performed utilizing diverse devices, including Mindray DC-80, Samsung HERA XW10, GE Voluson E10, and GE Logiq E9. All ultrasound scans were administered by licensed radiologists. If multiple images were obtained of a single mass, the image depicting the largest diameter was chosen for inclusion. Image quality control was carried out by two radiologists (T.W. and Y.L.).

### Construction of deep learning image segmentation model

A subset of images was randomly selected to train the segmentation model. Two investigators (L.L. and H.T.) used Labelme software (version 5.3.1, USA) to manually delineate the target lesions of the selected images and their segmentation results served as the standard. To avoid the subjective errors caused by individual differences, the interclass correlation coefficient (ICC) was utilized to assess the inter-observer or intra-observer concordance in the delineation of lesions. An ICC threshold of 0.75 or greater was deemed to reflect acceptable concordance.

Seven deep learning segmentation models were trained, including FCN ResNet50, FCN ResNet101, DeepLabV3 ResNet50, DeepLabV3 ResNet101, DeepLabV3 MobileNetV3-Large, LR-ASPP MobileNetV3-Large, and U-Net. We applied the dice similarity coefficient (DSC), which quantifies the overlap between the segmentation results of the investigator and the model, to evaluate the accuracy of segmentation. We chose the best model to automatically segment the remaining images. In cases where the segmentation was inaccurate, manual fine-tuning was performed.

### Features extraction and features selection

We used a feature analysis program designed for radiomics analysis within Pyradiomics to extract radiomic features. The extracted features were classified into three primary categories: geometry, intensity, and texture. The geometry feature depicts the shape characteristic of the lesion. The intensity feature illustrates the first-order statistical distribution of voxel intensity within the lesion. Texture features describe the pattern or the second- and high-order spatial distribution of the intensity, including gray-level co-occurrence matrix (GLCM), gray-level dependence matrix, gray-level size zone matrix, gray-level run-length matrix, and neighboring gray-tone difference matrix.

To identify features that exhibited the strongest correlation with the categorization, we employed the *t*-test and the Mann–Whitney *U*-test to screen features. We retained features associated with a *p* value less than 0.05, as they indicated significant differences between the two sets. To eliminate superfluous features, the correlation among the features was ascertained by calculating Spearman’s rank correlation coefficient. We selected only one feature from any pair presenting a correlation coefficient exceeding 0.9 to remove those exhibiting high redundancy. Additionally, we adopted a greedy recursive deletion approach to filter features, which involves the iterative removal of features deemed most superfluous in the current set.

We utilized the least absolute shrinkage and selection operator (LASSO) regression to minimize the feature set. The Rad scores were generated using LASSO logistic regression (LR), selecting only the features with non-zero coefficients. LASSO shrank all regression coefficients towards 0, depending on the regulation weight λ, and precisely set the coefficient of the uncorrelated feature to 0. The optimal λ value was ascertained through 10-fold cross-validation using minimum criteria, and the ultimate λ induced the least cross-validation error. The most robust feature with a non-zero coefficient was utilized for regression model fitting and integrated into a radiomic signature. A Rad score was generated from a linear combination of the selected features, each weighted according to the corresponding model coefficient.

### Model construction and evaluation

We utilized Python to establish radiomic and clinical models. The selected features were put into several machine learning algorithms, including LR, support vector machine (SVM), k-nearest neighbor (KNN), Random Forest, XGBoost, LightGBM, and multi-layer perception (MLP). We subsequently conducted a 5-fold cross-validation to ascertain the best hyperparameters for model fitting. A radiomic nomogram was developed, incorporating both radiomic features and clinical features for analysis.

The receiver operating characteristic (ROC) curve was used to visually assess the diagnostic performance. Furthermore, several diagnostic indices were calculated, such as the area under the ROC curve (AUC), specificity, sensitivity, accuracy, positive predictive value (PPV), negative predictive value (NPV), and precision. We performed the DeLong test to compare the AUCs of various models with MedCalc software. To evaluate the concordance between the model’s predictions and actual classifications, a calibration curve was generated to assess the calibration effectiveness using the Hosmer–Lemeshow analysis. The decision curve analysis (DCA) was performed to assess the clinical utility of the model.

### Assessment by radiologists

Two experienced radiologists (H.T. and W.C.) independently categorized the images based on O-RADS. A third experienced radiologist (L.L.) with more than ten years of gynecological ultrasound experience and two less-experienced radiologists (T.W. and Y.L.) with less than five years of gynecological ultrasound experience were tasked to categorize the images. With the assistance of the model, two less-experienced radiologists reclassified these images. In instances of uncertainty, less-experienced radiologists could refer to the nomogram results and the analysis of SHAP force plots for the images to potentially assist their diagnosis and adjust their classifications accordingly.

### Model interpretability and visualization

The SHAP method was used to visualize the significance of the features and their influence on the model, interpreting the internal decision-making process of the model by assigning importance values (SHAP values) to features.

### Statistical analysis

The IBM SPSS statistical software was employed to compare the clinical features of participants between the sets. The continuous variable was summarized using mean ± standard deviation and evaluated with the *t*-test (normal distribution) or Mann–Whitney *U*-test (non-normal distribution). The categorical variable was presented as a percentage and analyzed with the Chi-square test. A two-sided *p* value of less than 0.05 was considered statistically significant. The 95% confidence interval (CI) for the AUC was determined. Python was utilized to conduct the *Z* score normalization, Spearman rank correlation test, and LASSO analysis.

## Results

### Clinical features

**Fig. 2:**
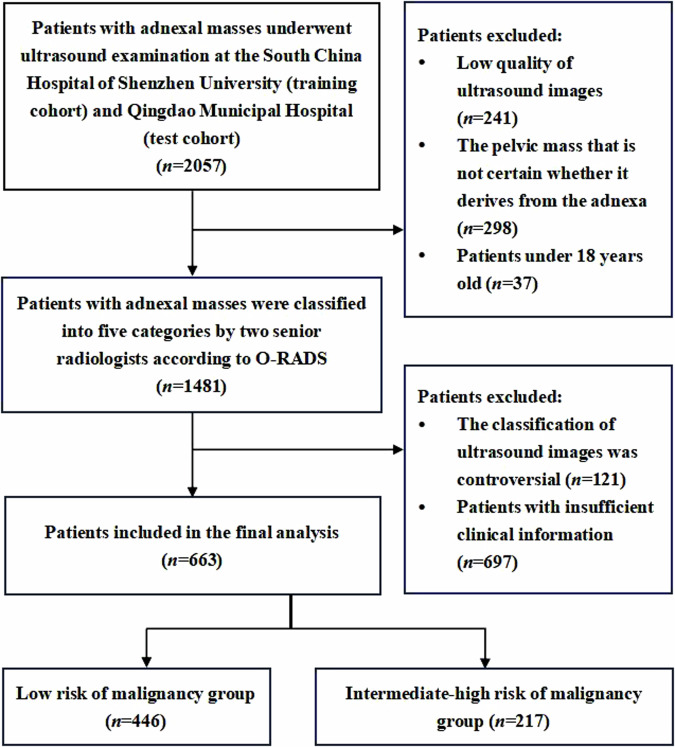
Flowchart of the study subjects screening based on inclusion and exclusion criteria

This study included a cohort of 663 participants with 663 adnexal masses. According to the classification by experienced radiologists, there were 446 cases in the low malignant risk set and 217 cases in the intermediate-high malignant risk set. The training set consisted of 530 cases, and the testing set comprised 133 cases, with an 8:2 ratio (Fig. 2). Significant differences were observed in all clinical features between the two sets in the training set (*p* < 0.05). However, in the testing set, only the age and proportion of symptomatic participants showed significant differences between the two sets (*p* < 0.05), while no significant differences were observed in the remaining clinical features (*p* > 0.05) (Table 1).

**Table 1:** Baseline clinical features of participants between two sets

Clinical features	Training all (*n* = 530)	Training low risk of malignancy set (*n* = 356)	Training intermediate-high risk of malignancy set (*n* = 174)	*p*	Testing all (*n* = 133)	Testing low risk of malignancy set (*n* = 90)	Testing intermediate-high risk of malignancy set (*n* = 43)	*p*
Age, (years)	34.88 ± 8.94	32.83 ± 8.10	39.06 ± 9.14	< 0.001	32.83 ± 7.25	31.73 ± 6.67	35.12 ± 7.94	0.030
Diameter, (mm)	41.24 ± 11.57	40.71 ± 12.08	42.31 ± 10.41	0.049	39.53 ± 10.89	39.41 ± 12.53	39.77 ± 6.31	0.287
Symptom				< 0.001				0.012
No	406 (76.60%)	254 (71.35%)	152 (87.36%)		105 (78.95%)	65 (72.22%)	40 (93.02%)	
Yes	124 (23.40%)	102 (28.65%)	22 (12.64%)		28 (21.05%)	25 (27.78%)	3 (6.98%)	
Menopause				< 0.001				0.063
No	482 (90.94%)	336 (94.38%)	146 (83.91%)		126 (94.74%)	88 (97.78%)	38 (88.37%)	
Yes	48 (9.06%)	20 (5.62%)	28 (16.09%)		7 (5.26%)	2 (2.22%)	5 (11.63%)	
Ascites				0.012				0.705
No	519 (97.92%)	353 (99.16%)	166 (95.40%)		132 (99.25%)	90 (100.00%)	42 (97.67%)	
Yes	11 (2.08%)	3 (0.84%)	8 (4.60%)		1 (0.75%)	0 (0%)	1 (2.33%)	

### Performance comparison of deep learning image segmentation models

A total of 204 images were randomly selected for manual annotation and segmentation model training. Among different models, the FCN ResNet101 demonstrated superior performance, achieving a DSC of 89.1% (Table 2). Consequently, the FCN ResNet101 was applied to automatically segment the remaining images. Notably, for masses predominantly composed of cystic lesions, the segmentation model achieved high accuracy (DSC: 92.3%). In contrast, the accuracy was somewhat lower for adnexal masses primarily consisting of solid lesions (DSC: 85.4%). Any imprecise segmentation was subsequently manually fine-tuned. On average, the segmentation process for an individual image took 6.3 s, including the time spent on manual fine-tuning. In comparison, manual annotation required an averaged of 13.6 s per image. The segmentation model facilitated a 53.7% reduction in the time required for lesion segmentation. Supplement Fig. 1 illustrates the segmentation outcomes generated by FCN ResNet101.

**Table 2:** DSC (%) of different deep learning-based segmentation models using the annotations from two investigators as the standard

Model	FCN ResNet50	FCN ResNet101	DeepLabV3 ResNet50	DeepLabV3 ResNet101	DeepLabV3 MobileNetV3-Large	LR-ASPP MobileNetV3-Large	U-Net
Investigator-1	87.7	89.1	86.7	88.1	83.3	75.0	48.6
Investigator-2	87.6	89.1	86.9	88.0	83.5	75.0	48.7

### Features extraction and selection

**Fig. 3:**
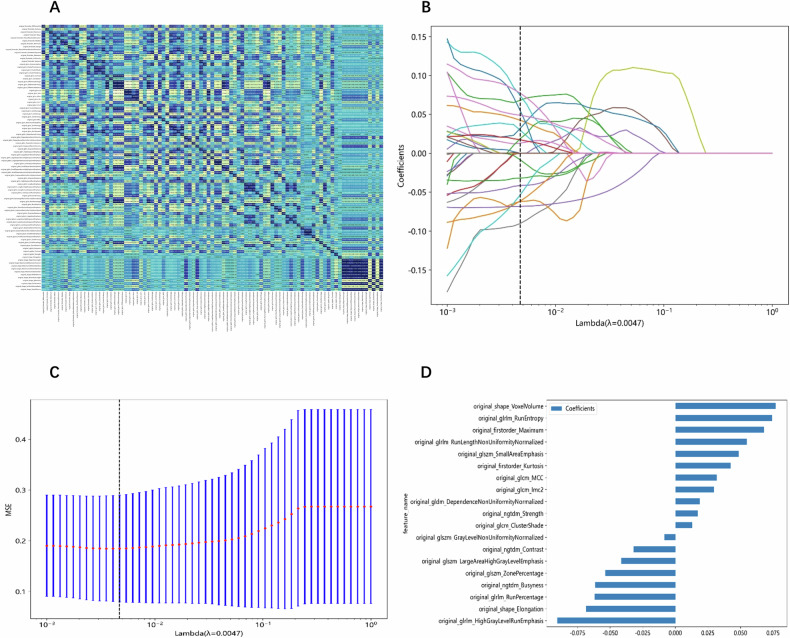
Feature extraction and selection. **A** Spearman correlation coefficient between each feature. **B** Coefficients of 10-fold cross-validation based on LASSO algorithm. **C** MSE of 10-fold cross-validation based on LASSO algorithm. **D** Histogram depicting the values of coefficients in the final selected non-zero features. MSE, mean square error

A total of 107 handcrafted features were extracted (shape feature: 14, first-order feature: 18, texture feature: 75). A LASSO regression was employed to choose 19 features with non-zero coefficients for constructing the Rad score. Information on the feature extraction and selection process is presented in Fig. 3.

### Performance of the radiomic model

**Fig. 4:**
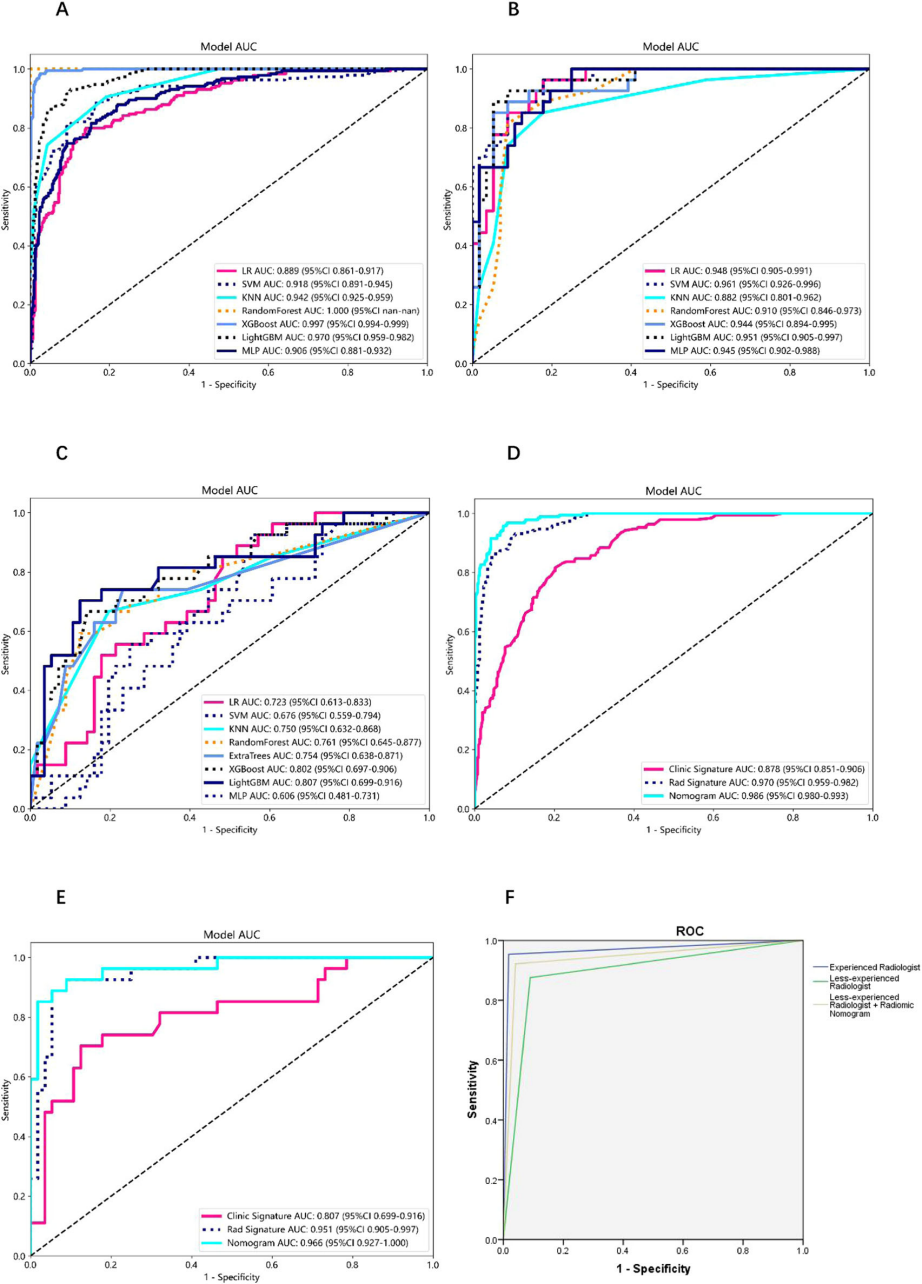
The ROC curves and AUC of different models. **A** The ROC curves of different radiomic models in the training set. **B** The ROC curves of different radiomic models in the testing set. **C** The ROC curves of different clinical models in the testing set. **D** The ROC curves of the clinical, radiomic, and nomogram models in the training set. **E** The ROC curves of the clinical, radiomic, and nomogram models in the testing set. **F** The ROC curves for experienced radiologists and less-experienced radiologists without and with the assistance of the radiomic nomogram

Seven machine learning models were developed and compared to determine the best one for classification (Table 3). In training set, Random Forest achieved the highest performance (AUC: 1.000, 95% CI: 1.000–1.000, accuracy: 99.0%, sensitivity: 96.8%, specificity: 100.0%, PPV: 100.0%, NPV: 98.5%, precision: 100.0%) (Fig. 4A). In testing set, SVM achieved the best performance (AUC: 0.961, 95% CI: 0.925–0.996, accuracy: 89.2%, sensitivity: 85.2%, specificity: 91.1%, PPV: 82.1%, NPV: 92.7%, precision: 82.1%) (Fig. 4B).

**Table 3:** Diagnostic performance of different radiomic models for classification

Cohort	Model	AUC (95% CI)	Accuracy, (%)	Sensitivity, (%)	Specificity, (%)	PPV, (%)	NPV, (%)	Precision, (%)
Training	LR	0.889 (0.861–0.917)	83.8	79.5	85.9	73.3	89.6	73.3
Training	SVM	0.918 (0.891–0.945)	85.2	87.9	83.8	72.6	93.4	72.6
Training	KNN	0.942 (0.925–0.959)	88.6	74.2	95.6	89.2	88.4	89.2
Training	Random Forest	1.000 (1.000–1.000)	99.0	96.8	100.0	100.0	98.5	100.0
Training	XGBoost	0.997 (0.994–0.999)	97.6	97.9	97.4	94.9	99.0	94.9
Training	LightGBM	0.970 (0.959–0.982)	90.7	92.6	89.7	81.5	96.2	81.5
Training	MLP	0.906 (0.881–0.932)	83.4	81.1	84.6	72.0	90.2	72.0
Testing	LR	0.948 (0.905–0.991)	85.5	92.6	82.1	71.4	95.8	71.4
Testing	SVM	0.961 (0.926–0.996)	89.2	85.2	91.1	82.1	92.7	82.1
Testing	KNN	0.882 (0.801–0.962)	85.5	74.1	91.1	80.0	87.9	80.0
Testing	Random Forest	0.910 (0.846–0.973)	81.9	59.3	92.9	80.0	82.5	80.0
Testing	XGBoost	0.944 (0.894–0.995)	89.2	85.2	91.1	82.1	92.7	82.1
Testing	LightGBM	0.951 (0.905–0.997)	90.4	88.9	91.1	82.8	94.4	82.8
Testing	MLP	0.945 (0.902–0.988)	81.9	96.3	75.0	65.0	97.7	65.0

### Performance of the radiomic model, clinical model, and nomogram

**Fig. 5:**
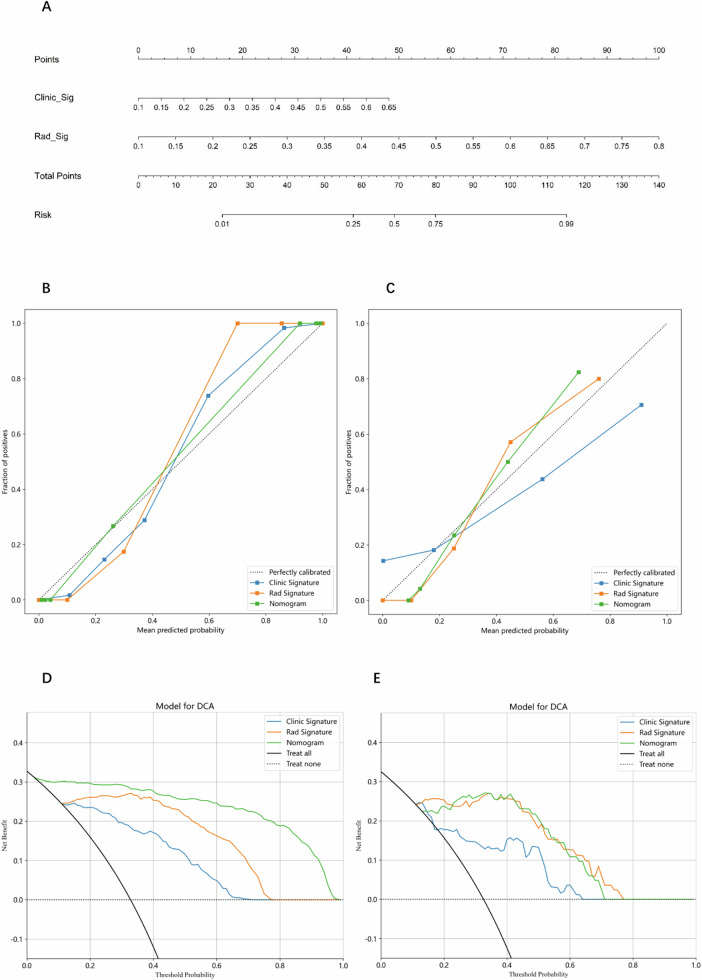
**A** The nomogram with a total score reflecting the probability of malignancy in adnexal masses. **B** The calibration curves for clinical, radiomic, and nomogram models in the training set. **C** The calibration curves for clinical, radiomic, and nomogram models in the testing set. **D** The DCA curves for clinical, radiomic, and nomogram models in the training set. **E** The DCA curves for clinical, radiomic, and nomogram models in the testing set

Clinical features that showed significant differences between the two sets in the training set were selected to establish the clinical model. The LightGBM algorithm exhibited the highest performance in testing set (AUC: 0.807, 95% CI: 0.699–0.916, accuracy: 80.7%, sensitivity: 66.7%, specificity: 87.5%, PPV: 72.0%, NPV: 84.5%, and precision: 72.0%) (Fig. 4C). The nomogram with LightGBM model exhibited the highest diagnostic performance in testing set (AUC: 0.966, 95% CI: 0.927–1.000, accuracy: 90.4%, sensitivity: 88.9%, specificity: 91.1%, PPV: 82.8%, NPV: 94.4%, precision: 82.8%). Figure 5A depicts the nomogram, which calculates a total score that reflects the likelihood of malignancy. The diagnostic indices for the radiomic model, clinical model, and nomogram are presented in Table 4 and Fig. 4D, E.

**Table 4:** Diagnostic performance of radiomic, clinical, and nomogram models for classification

Cohort	Model	AUC (95% CI)	Accuracy, (%)	Sensitivity, (%)	Specificity, (%)	PPV, (%)	NPV, (%)	Precision, (%)
Training	Clinical	0.878 (0.851–0.906)	80.2	81.1	79.7	66.1	89.6	66.1
Training	Radiomic	0.970 (0.959–0.982)	90.7	92.6	89.7	81.5	96.2	81.5
Training	Nomogram	0.986 (0.980–0.993)	93.3	96.3	91.8	85.1	98.1	85.1
Testing	Clinical	0.807 (0.699–0.916)	80.7	66.7	87.5	72.0	84.5	72.0
Testing	Radiomic	0.951 (0.905–0.997)	90.4	88.9	91.1	82.8	94.4	82.8
Testing	Nomogram	0.966 (0.927–1.000)	90.4	88.9	91.1	82.8	94.4	82.8

The DeLong test demonstrated the comparison of the AUC between the nomogram and clinical model, as well as between the nomogram and radiomic model, was of statistical significance (*p* < 0.05) in both training and testing sets. The Hosmer–Lemeshow test exhibited good concordance between the nomogram’s predictions and the true classifications in training and testing sets (*p* > 0.05). Conversely, the predictions made by the clinical or radiomic models did not align as well with the actual classifications (*p* < 0.05) (Table 5 and Fig. 5B, C), indicating the nomogram fits perfectly. The DCA curve demonstrated both the nomogram and radiomic model substantially enhanced the intervention results for patients (Fig. 5D, E).

**Table 5:** *p* value of Hosmer–Lemeshow test

Cohort	Clinical model	Radiomic model	Nomogram
Training	< 0.001	< 0.001	0.053
Testing	< 0.001	< 0.001	0.219

### Performance of less-experienced radiologists

The classification performance of the radiomic nomogram was comparable to that of the experienced radiologist (AUC: 0.968, 95% CI: 0.950–0.986, sensitivity: 95.4%, specificity: 98.2%) (*p* > 0.05). However, the diagnostic efficiency of the less-experienced radiologists was inferior to that of the nomogram (average AUC: 0.893, 95% CI: 0.863–0.923, sensitivity: 87.6%, specificity: 91.0%) (*p* < 0.05). Nevertheless, the assistance of the nomogram significantly enhanced the diagnostic efficiency of less-experienced radiologists (average AUC: 0.941, 95% CI: 0.917–0.964, sensitivity: 92.2%, specificity: 96.0%) (Fig. 4F).

### Model interpretability and visualization

**Fig. 6:**
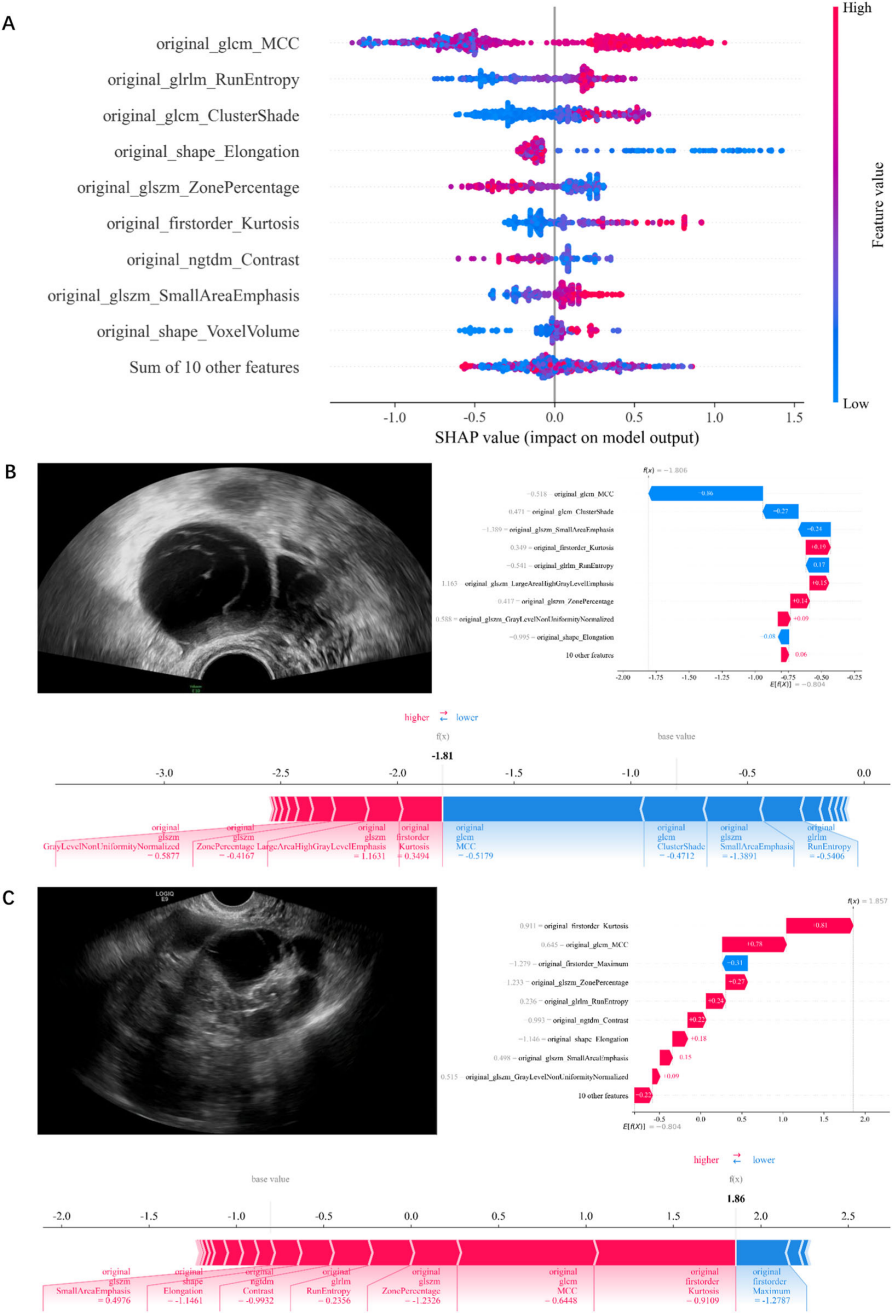
**A** The SHAP summary plot of the nomogram. **B** The SHAP force plot for an image from the low malignant risk set: the final SHAP value for this image is −1.806, which is lower than the base value of −0.804, thereby classifying the mass as a low malignant risk lesion by the nomogram. **C** The SHAP force plot for an image from the intermediate-high malignant risk set: the final SHAP value for this image is 1.857, which is larger than the base value of −0.804, thereby classifying the mass as an intermediate-high malignant risk lesion by the nomogram

The SHAP method offers interpretability and visualization for the nomogram. The SHAP summary plot provides a global interpretation of the model by visualizing the importance of features through SHAP values (Fig. 6A). Each dot represents an individual sample, and its SHAP value indicates the importance of the corresponding feature, with the larger absolute value of the SHAP value signifying greater contribution to the model’s prediction outcome. The color of the dot represents the impact of the feature value on model predictions. The most important feature is positioned at the top of the plot, indicating that the original_glcm_MCC is the most significant feature for the nomogram in this study.

The SHAP force plot is designed to interpret and visualize the prediction outcomes for individual samples (Fig. 6B, C). The SHAP values of the features for an individual image and their contributions to the model are depicted by colored arrows. The length of the arrow represents the extent of the feature’s impact on the final prediction value, with longer arrows indicating greater contributions; the color of the arrow distinguishes between positive contributions (red) to the prediction results and negative ones (blue). The final SHAP value, which is the summation of the base value and the cumulative contributions from all features, represents the model’s ultimate prediction for the image.

## Discussion

The segmentation of adnexal masses is a crucial preliminary stage in the development of a classification model. Nonetheless, manual delineation is a time-consuming and laborious task. The adnexa are situated within the pelvic cavity, nestled deep in the intestinal canal, which often results in unclear boundaries between the adnexa and adjacent organs. Additionally, inter-observer variability in manual annotation can arise from differences in subjectivity among radiologists. To reduce reliance on manual annotation and enhance the efficiency of model development, we used AI techniques for the automation of segmentation. We trained seven deep learning-based models that have been extensively adopted in previous literature [23–25] to identify the optimal model. The FCN ResNet101 achieved the highest performance and reduced the time by 53.7%, significantly enhancing efficiency. Previous studies on segmentation were predominantly based on CT images. Compared to prior studies based on ultrasound images [26, 27], this study achieved a slightly higher segmentation accuracy. Furthermore, this model exhibited greater accuracy when segmenting adnexal masses primarily composed of cystic lesions, as opposed to those mainly composed of solid lesions. This discrepancy can be attributed to the typical anechoic appearance of cystic lesions, which starkly contrasts the echogenicity of surrounding tissues. Conversely, solid lesions often exhibit boundary uncertainties due to their similar echogenicity to neighboring organs.

The O-RADS system allows for the categorization of adnexal lesions according to the morphological characteristics of ultrasound, thereby indicating the potential malignancy risk associated with each type [15]. It has been validated in previous studies and has demonstrated good inter-reader concordance [16–19]. In this study, we constructed a nomogram combining radiomic and clinical features to automate the categorization of adnexal masses. In accordance with published studies and expert consensus endorsing pattern recognition by senior radiologists as the most precise ultrasonic approach for distinguishing adnexal masses [28–30], we utilized the assessments of experienced radiologists as our benchmark to assess diagnostic performance. Our findings revealed the fusion model demonstrated enhanced diagnostic performance compared to either radiomic or clinical models alone, implying the integration of these features is especially beneficial for differentiating benign from malignant masses. The DeLong test confirmed the fusion model significantly outperformed both clinical and radiomic models for the classification. Calibration curves demonstrated satisfactory concordance between the fusion model’s predictions and the true classifications. The DCA results indicated that the application of the model provides significant clinical advantages in comparison with situations where no predictive model is employed.

The diagnostic performance of the nomogram was comparable to that of experienced radiologists and outperformed less-experienced radiologists. Furthermore, the assistance of the nomogram significantly enhanced the diagnostic efficiency of less-experienced radiologists, potentially narrowing the disparity in medical resources. The primary reasons for classification errors among less-experienced radiologists included: (1) less-experienced radiologists confused the internal echoes of benign lesions (such as blood products in hemorrhagic cyst or endometrioma, and dermoid contents in dermoid cyst) with the solid component echoes of malignant lesions, leading to incorrect classifications. (2) Bilocular and multilocular cysts with irregular inner walls, or cysts with small papillary projections, which should have been categorized as O-RADS 4–5, were mistakenly classified as O-RADS 3. (3) According to the O-RADS Ultrasound version 2022 [15], shadowing for solid lesions with smooth contours was added as a morphologic feature favoring benignity. Consequently, smooth solid lesions with shadowing are now classified as O-RADS 3, in distinction to previous scoring as O-RADS 4. However, less-experienced radiologists still misclassified these masses as O-RAD 4.

The black-box characteristic of machine learning algorithms significantly impedes the widespread adoption of AI models [31]. In this study, we innovatively employed the SHAP method to achieve the interpretability and visualization of the model decision-making process. The SHAP summary plot provides a clinician-friendly illustration of the significance of features and their impact on the model [32]. The SHAP force plot enables us to comprehend the contributions of the features of each individual image to the classification result. This method is highly useful in the training of radiological staff and potentially assists in improving their professional skills. In instances of diagnostic difficulty or uncertainty, less-experienced radiologists could refer to the analysis of SHAP force plots to understand the model’s decision-making process and identify which features (shape, texture, or intensity) were most crucial in the classification process.

In clinical practice, accurate classification for adnexal mass remains challenging, particularly in regions with a shortage of experienced radiologists. The models hold the potential to be integrated into routine clinical practice and reshape diagnostic pathways. Before diagnosis by radiologists, these models can serve as preliminary screening tools for malignant lesions, significantly alleviating the workload of radiologists. Additionally, the models can also assist junior radiologists in improving their professional skills by visualizing the diagnostic process of the models through SHAP.

The retrospective nature of the study posed challenges in collecting adequate clinical data, which led to suboptimal diagnostic efficiency of the clinical model. The intermediate-high malignant risk set had an older average age and a greater percentage of postmenopausal participants compared to the low malignant risk set, aligning with the findings of previous research [33, 34]. The majority of ovarian cancer cases either show no symptoms or exhibit nonspecific symptoms [35, 36]. Within the low malignant risk set, patients with endometriosis were observed to frequently present with symptoms such as dysmenorrhea or chronic pelvic pain, which resulted in an elevated incidence of clinical symptoms.

Radiomics enables the transformation of imaging data into high-throughput, analyzable image features through the use of advanced image processing techniques, and it has been widely adopted in medical image analysis [37, 38]. Advanced AI algorithms are used to analyze the image features that link to pathological diagnoses [39]. Multiple studies have been published on the use of AI models for diagnosing adnexal masses [22, 40–42] and exhibited commendable classification abilities. For instance, Gao et al [43], Zhang et al [44], and Wang et al [45] developed models that achieved superior accuracy rates in differentiating between benign and malignant ovarian lesions compared to radiologists. However, these studies have concentrated on differentiating lesions based on pathological outcomes. To our knowledge, the exploration of AI applications for classifying adnexal masses based on O-RADS remains a scarcely explored area.

The limitations are outlined below. Firstly, the restricted sample size of the dataset from the dual-center may introduce potential selection bias, which limits the generalizability of the results. Secondly, due to the retrospective nature of this study, the limited availability of clinical data constrained the diagnostic efficacy of the clinical model. Future prospective, multicenter studies involving larger populations and comprehensive clinical datasets are warranted to validate the model’s diagnostic accuracy in clinical settings and enhance the generalizability of the model.

## Conclusions

The segmentation model serves as a valuable instrument for the automated segmentation of adnexal lesions. The machine learning model has achieved commendable performance in classifying the adnexal masses into low malignant risk and intermediate-high malignant risk masses based on O-RADS, and it holds the potential to enhance the skill level of less-experienced radiologists. Furthermore, this model offers an effective screening approach for ovarian cancer and can supply additional valuable clinical insights that help in the development of therapeutic strategies for adnexal lesions.

## Supplementary information

ELECTRONIC SUPPLEMENTARY MATERIAL

## Data Availability

The data presented in this study are available on request from the corresponding author (W.Z.). The data are not publicly available due to hospital regulations.

## Abbreviations

AI: Artificial intelligence
AUC: Area under curve
CI: Confidence interval
DCA: Decision curve analysis
DSC: Dice similarity coefficient
ICC: Interclass correlation coefficient
KNN: k-Nearest neighbor
LASSO: Least absolute shrinkage and selection operator
LR: Logistic regression
MLP: Multi-layer perception
NPV: Negative predictive value
O-RADS: Ovarian-adnexal reporting and data system
PPV: Positive predictive value
ROC: Receiver operating characteristic
SHAP: SHapley Additive exPlanations
SVM: Support vector machine
