# Supplementary material for: Automatic segmentation model and machine learning model grounded in ultrasound radiomics for distinguishing between low malignant risk and intermediate-high malignant risk of adnexal masses
Source: Insights Imaging. 2025 Jan 13;16:14. doi: 10.1186/s13244-024-01874-7 (PMC11729609; doi:10.1186/s13244-024-01874-7)
Supplement: Supplementary file 1 — ELECTRONIC SUPPLEMENTARY MATERIAL [file 13244_2024_1874_MOESM1_ESM.pdf]

Automatic segmentation model and machine learning model grounded in ultrasound radiomics for distinguishing between low malignant risk and intermediate-high malignant risk of adnexal masses

ELECTRONIC SUPPLEMENTARY MATERIAL

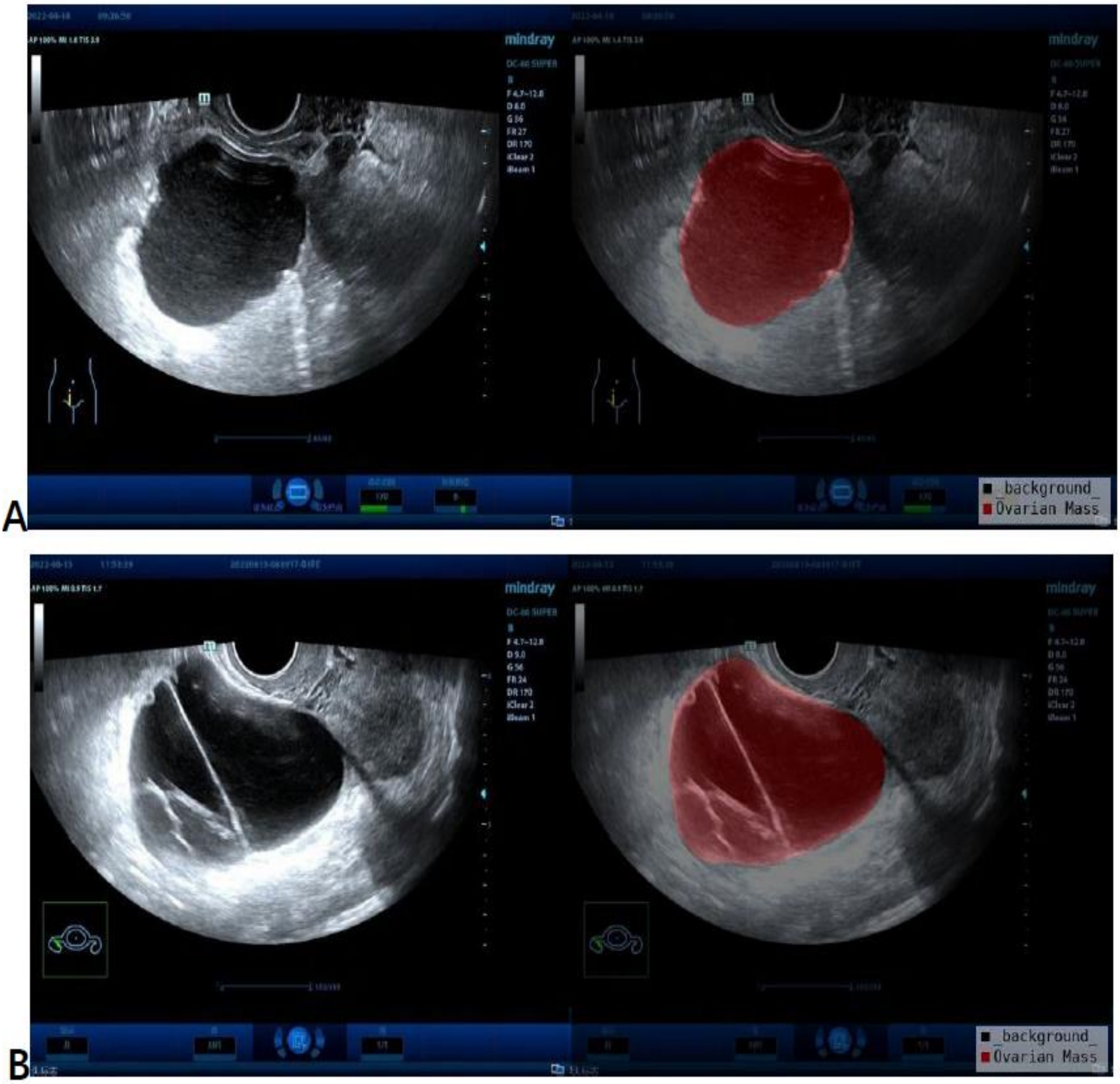

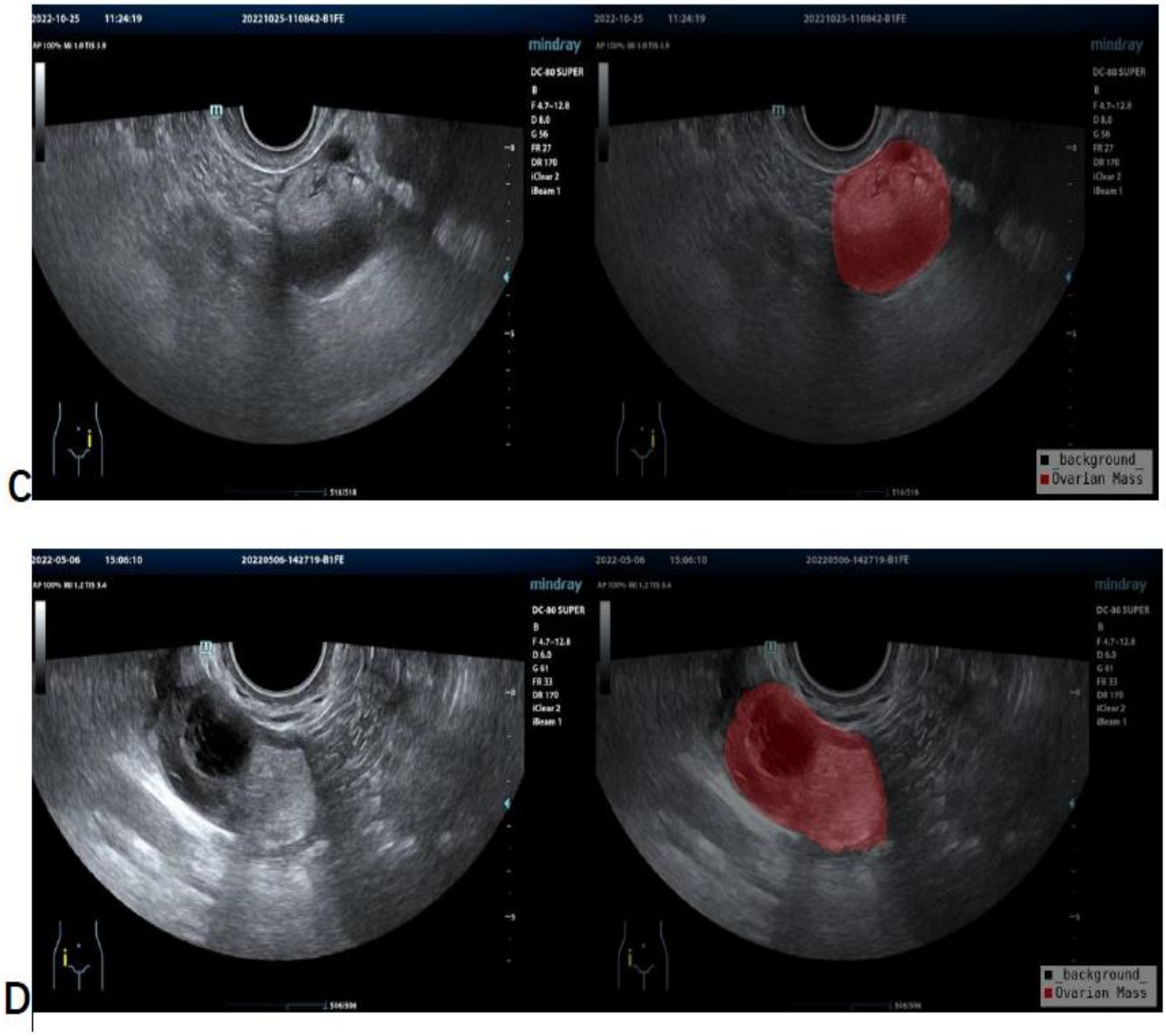

Supplement Figure 1. The segmentation results of the automatic segmentation model FCN ResNet101. (A-B) For adnexal masses mainly composed of cystic lesions, the automatic segmentation model achieved high accuracy. (C-D) For adnexal masses mainly composed of solid lesions, the accuracy of the automatic segmentation model was relatively low.
